# Breaking the Storm: Hemophagocytic Lymphohistiocytosis/Macrophage Activation Syndrome as the First Manifestation of Systemic Lupus Erythematosus/Mixed Connective Tissue Disease in an Adolescent Successfully Treated With Anakinra

**DOI:** 10.7759/cureus.116818

**Published:** 2026-09-24

**Authors:** Ramia Alhasan, Ahmad T Alenizi, Mahmoud Adly, Mustafa Mahmoud, Mohamed Tharwat Hegazy, Samer Alhasan

**Affiliations:** 1 Rheumatology Department, Jahra Hospital, Jahra, KWT; 2 Internal Medicine Department, Jahra Hospital, Jahra, KWT

**Keywords:** autoimmune connective tissue disease, hemophagocytic lymphohistiocytosis (hlh), macrophage activation syndrome (mas), serum ferritin, systemic lupus erythematosus (sle)

## Abstract

Macrophage activation syndrome (MAS), a form of secondary hemophagocytic lymphohistiocytosis (HLH), is an uncommon but life-threatening complication of autoimmune disease and may occasionally represent the first clinical manifestation of systemic lupus erythematosus (SLE). We describe the case of a previously healthy 15-year-old girl who initially presented with persistent fever, mucocutaneous ulcerations, fatigue, and pancytopenia. Her evaluation revealed low complement levels, elevated lactate dehydrogenase, and splenomegaly, and subsequent serologic testing confirmed new-onset SLE with features of mixed connective tissue disease and Class II lupus nephritis. Although she showed early improvement after receiving corticosteroids and hydroxychloroquine, her condition unexpectedly deteriorated with rising ferritin, hypertriglyceridemia, worsening cytopenias, and progressive liver enzyme elevation, raising concern for an evolving hyperinflammatory syndrome. She ultimately fulfilled five HLH-2004 criteria and had an HScore of 261, indicating a very high probability of HLH/MAS, despite the absence of hemophagocytosis on bone marrow biopsy. Targeted immunomodulatory therapy with anakinra was initiated promptly and resulted in rapid clinical and biochemical improvement. This case highlights the diagnostic challenges of MAS when it emerges in parallel with newly diagnosed SLE, emphasizes the importance of distinguishing MAS from SLE flare or infection, and introduces interleukin-1 blockade as a potential therapeutic option. Early recognition and timely introduction of targeted anti-inflammatory therapy may contribute to rapid stabilization and to be lifesaving when corticosteroids alone are insufficient to halt the cytokine-driven inflammatory cascade.

## Introduction

Macrophage activation syndrome (MAS) is a severe hyperinflammatory condition marked by uncontrolled activation of macrophages and cytotoxic T cells, resulting in excessive cytokine release, cytopenias, organ dysfunction, and, if untreated, significant mortality [1]. Although MAS is well recognized in systemic juvenile idiopathic arthritis, it also occurs in systemic lupus erythematosus (SLE), often complicating active disease or infection [2].

MAS is considered a form of secondary hemophagocytic lymphohistiocytosis (HLH), and the two terms are closely related. MAS as the initial presentation of an SLE flare or infection is clinically critical, as delayed recognition may prevent timely initiation of lifesaving therapy. In this context, interleukin-1 (IL-1) blockade has emerged as a potential option for severe or refractory MAS. MAS as the initial presentation of SLE remains rare, particularly in adolescents, and can easily be overlooked because many early clinical and laboratory features overlap with active lupus or infectious etiologies [3].

Diagnosis is further complicated by the sometimes discordant inflammatory profile seen in MAS. Despite severe systemic inflammation, the erythrocyte sedimentation rate (ESR) may fall as fibrinogen levels decline. In this setting, serial trends in ferritin, fibrinogen, triglycerides, and cytopenias provide important diagnostic clues [4]. The HScore, which incorporates clinical, laboratory, and bone marrow parameters, can provide additional diagnostic support in suspected secondary hemophagocytic syndromes, particularly when bone marrow findings are nondiagnostic [5].

We present a case of MAS as the first manifestation of SLE/mixed connective tissue disease (MCTD) in a previously healthy adolescent. Her rapid clinical deterioration despite initial immunosuppressive therapy and subsequent dramatic improvement following anakinra highlight the importance of early recognition and timely targeted treatment [6].

## Case presentation

A 15-year-old previously healthy girl presented with a 10-day history of high-grade fever and upper respiratory tract symptoms. Before admission, she had received oral amoxicillin-clavulanate without improvement and subsequently developed severe stomatitis, painful oral ulcerations, crusted erythematous lesions involving the lips and external ear, progressive fatigue, and persistent fever. On day one, she was admitted to Al-Jahra Hospital. She appeared acutely unwell and fatigued, with marked mucositis, crusted lesions involving the upper lip and external ear (Figure 1), and cervical lymphadenopathy; however, she remained hemodynamically stable.

**Figure 1:**
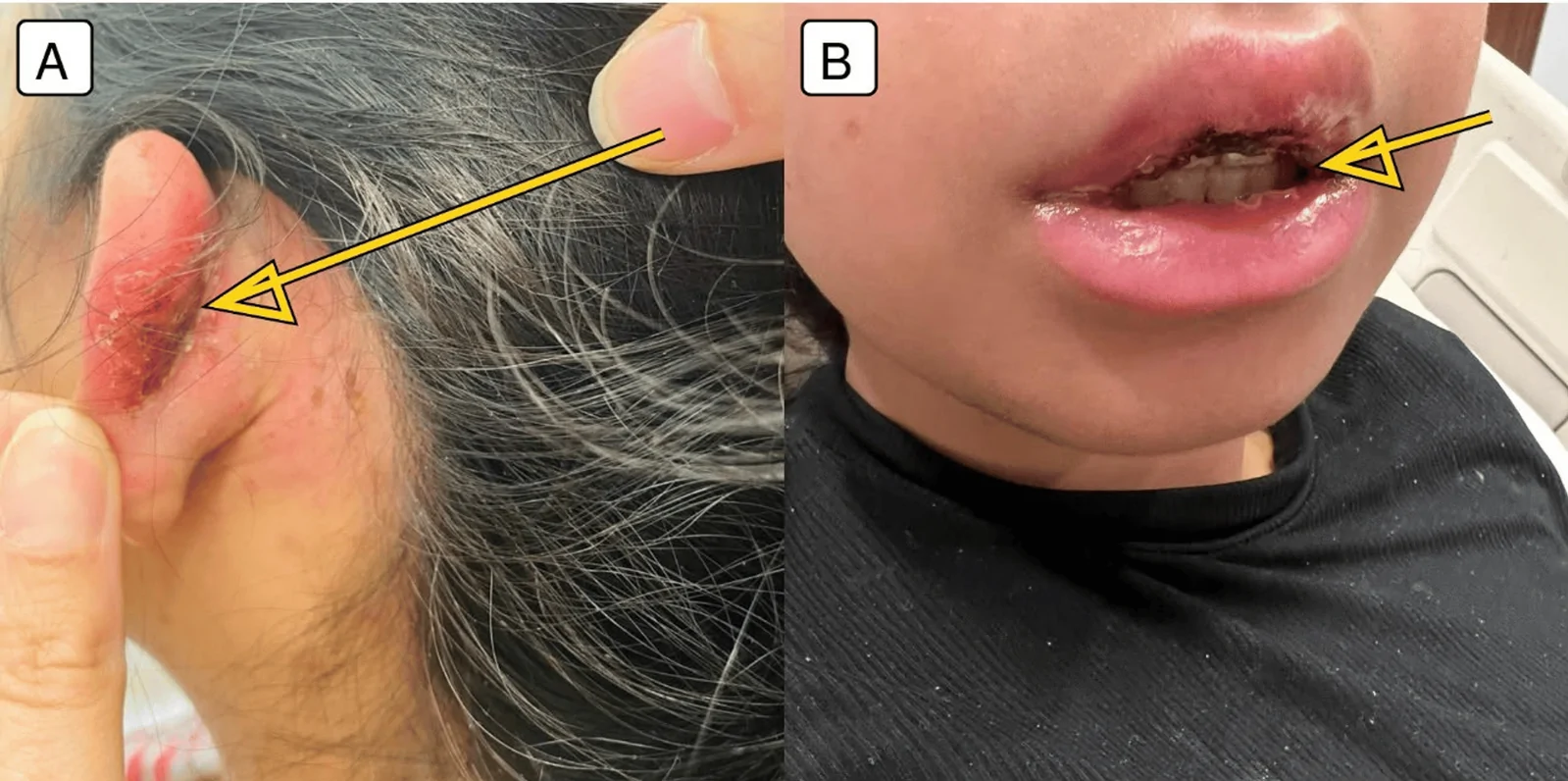
Mucocutaneous manifestations at presentation. (A) Crusted erythematous lesion involving the external ear. (B) Crusted ulcerative lesion involving the upper lip. Yellow arrows indicate the affected areas.

On day one, initial laboratory investigations demonstrated pancytopenia, with a white blood cell count of 2.0 × 10⁹/L, hemoglobin of 88 g/L, and platelet count of 92 × 10⁹/L. The reticulocyte count was reduced, suggesting an inadequate bone marrow response. Despite the severity of her presentation, C-reactive protein and procalcitonin were low, while the ESR was mildly elevated. Serum lactate dehydrogenase (LDH) was elevated, with mild transaminitis and a ferritin level of 213 µg/L. Complement C3 and C4 levels were reduced, whereas renal function remained normal. Peripheral blood smear showed no abnormal cells. Chest radiography was normal, while abdominal ultrasonography demonstrated splenomegaly measuring 15.5 cm. The principal laboratory trends during hospitalization are summarized in Table 1.

**Table 1:** Serial laboratory findings during hospitalization and following anakinra initiation. LDH = lactate dehydrogenase; AST = aspartate aminotransferase; ALT = alanine aminotransferase; TG = triglyceride; PLT = platelet count; Hb = hemoglobin; WBC = white blood cell count

LDH (90–248 U/L)	AST/ALT (0–35 U/L)	TG (0.4–1.75 mmol/L)	Ferritin (ng/mL)	PLT (150–410 × 10⁹/L)	Hb (120–150 g/L)	WBC (4–10 × 10⁹/L)	Hospital day (date)
446	—	—	213.7	80	88	2.6	Day 1
733	172/55	—	791	90	82	1.9	Day 8
573	140/42	—	2,145	136	72	2.4	Day 15
1,053	356/103	—	—	—	—	—	Day 17
922	223/93	5.61	3,649	—	—	—	Day 18
—	—	—	1,535	175	64	4.4	Day 20
—	—	—	476.5	—	—	—	Day 25

During days 2-14, her fever persisted, and the mucocutaneous lesions worsened despite empiric antimicrobial therapy. Extensive infectious testing, including viral serologies, was negative. In view of the mucocutaneous ulcerations, cytopenias, hypocomplementemia, and newly detected proteinuria, an autoimmune evaluation was undertaken. Antinuclear antibodies and anti-dsDNA antibodies were strongly positive, while the extractable nuclear antigen profile supported features of MCTD. Renal biopsy demonstrated Class II lupus nephritis, establishing the diagnosis of new-onset SLE with overlapping features of MCTD. Intravenous methylprednisolone 40 mg daily and hydroxychloroquine were initiated, resulting in initial improvement in fever, stomatitis, and overall energy.

On day 15, the patient developed recurrent fever and increasing fatigue despite corticosteroid therapy. Ferritin had increased to 2,145 ng/mL, with persistent cytopenias and progressive liver enzyme elevation. By days 17-18, her clinical and biochemical condition had deteriorated further: ferritin reached 3,649 ng/mL, triglycerides increased to 5.61 mmol/L, aspartate aminotransferase peaked at 356 U/L, alanine aminotransferase at 103 U/L, and LDH at 1,053 U/L. She also developed bilateral hand tremor; however, brain MRI was unremarkable, and the tremor was attributed to corticosteroid exposure. This evolving pattern raised strong suspicion for MAS.

On day 18, bone marrow examination showed no evidence of hemophagocytosis. Nevertheless, the absence of this finding did not exclude MAS because of its limited diagnostic sensitivity. The patient fulfilled five HLH-2004 criteria: fever, splenomegaly, cytopenias, hyperferritinemia, and hypertriglyceridemia. Her HScore was 261, corresponding to a very high probability of reactive hemophagocytic syndrome.

High-dose methylprednisolone pulses (500 mg daily for three days) were administered; however, due to the severity of her hyperinflammatory state, targeted therapy with anakinra (60 mg subcutaneously once daily) was initiated promptly. Early targeted treatment with cytokine-directed therapy is supported by contemporary HLH/MAS consensus guidance, although the evidence for anakinra remains largely observational. Within 48 hours of anakinra initiation, she showed rapid clinical and biochemical improvement. Fever resolved completely, stomatitis and skin lesions regressed, appetite and energy improved, and laboratory parameters normalized steadily, including ferritin falling to 1,500 µg/L and LDH decreasing to 550 U/L.

After stabilization, she was transitioned to oral prednisolone 40 mg daily with taper, mycophenolate mofetil 1 g twice daily, hydroxychloroquine 200 mg daily, and continued anakinra. She also received valacyclovir prophylaxis and gastric and bone-protective therapy. She was discharged in stable condition with continued outpatient follow-up showing sustained improvement.

## Discussion

This case illustrates the diagnostic complexity of identifying MAS when it presents concurrently with new-onset SLE, especially in adolescents without prior medical conditions. Both conditions share overlapping clinical features, including fever, cytopenias, mucocutaneous involvement, and elevated LDH. In early stages, MAS may mimic uncontrolled lupus activity or infection [7]. The patient’s normal C-reactive protein and procalcitonin were misleadingly reassuring [8], but such findings may occur in MAS and should not delay recognition when other clinical and laboratory features suggest hyperinflammation [9].

Trend-based laboratory interpretation proved critical. The rapid rise in ferritin was a key early warning sign of hyperinflammation, particularly when accompanied by rising triglycerides, worsening liver enzymes, and progressive cytopenias [10]. These abnormalities, along with her splenomegaly and neurological symptoms, shifted suspicion toward MAS [10]. The HScore provided objective support, with a score of 261 indicating a very high probability of reactive hemophagocytic syndrome in the original validation study [11]. Notably, the absence of hemophagocytosis on bone marrow biopsy did not exclude MAS, as hemophagocytosis may be absent and is neither sufficiently sensitive nor specific to establish or exclude the diagnosis [12,13].

The patient’s rapid clinical and laboratory improvement following anakinra underscores the potential value of early targeted therapy in severe MAS. Anakinra, a recombinant IL-1 receptor antagonist, is increasingly used as an immunomodulatory therapy in secondary HLH/MAS, particularly in rheumatologic disease, and is included among early empiric treatment options in current European Alliance of Associations for Rheumatology (EULAR)/American College of Rheumatology (ACR) guidance [10]. However, the evidence supporting its use remains largely derived from case reports, case series, and observational experience, with systematic reviews synthesizing these predominantly non-randomized data [14,15]. In our patient, the close temporal association between initiation of anakinra and rapid clinical improvement after an inadequate response to corticosteroids supports a potential therapeutic contribution of IL-1 blockade, although causality cannot be established from a single case.

The coexistence of newly diagnosed SLE/MCTD and MAS also emphasizes the importance of treating the underlying autoimmune disease while simultaneously controlling the hyperinflammatory syndrome. Current EULAR recommendations support hydroxychloroquine as foundational therapy for SLE and individualized use of immunosuppressive therapy according to organ involvement and disease severity [16].

## Conclusions

HLH/MAS can be the first manifestation of an underlying autoimmune disease such as SLE/MCTD, particularly in adolescents. Early recognition depends on careful interpretation of evolving laboratory trends rather than isolated values. Rising ferritin, cytopenias, hypertriglyceridemia, and persistent fever despite immunosuppression should prompt urgent evaluation for MAS. The HScore is a valuable tool when diagnosis is uncertain. Early initiation of targeted anti-inflammatory therapy, including anakinra in appropriate patients, in combination with high-dose corticosteroids may contribute to rapid stabilization and improved outcomes. This case emphasizes that causality cannot be established from a single case and also emphasizes the need for heightened clinical vigilance when managing young patients with newly diagnosed autoimmune disease who exhibit signs of systemic hyperinflammation.

## References

[1] Ravelli A, Grom AA, Behrens EM, Cron RQ (2012). Macrophage activation syndrome as part of systemic juvenile idiopathic arthritis: diagnosis, genetics, pathophysiology and treatment. Genes Immun.

[2] Abdirakhmanova A, Sazonov V, Mukusheva Z, Assylbekova M, Abdukhakimova D, Poddighe D (2021). Macrophage activation syndrome in pediatric systemic lupus erythematosus: a systematic review of the diagnostic aspects. Front Med (Lausanne).

[3] Pringe A, Trail L, Ruperto N (2007). Macrophage activation syndrome in juvenile systemic lupus erythematosus: an under-recognized complication?. Lupus.

[4] Yeap ST, Sheen JM, Kuo HC, Hwang KP, Yang KD, Yu HR (2008). Macrophage activation syndrome as initial presentation of systemic lupus erythematosus. Pediatr Neonatol.

[5] Fardet L, Galicier L, Lambotte O (2014). Development and validation of the HScore, a score for the diagnosis of reactive hemophagocytic syndrome. Arthritis Rheumatol.

[6] Charlesworth JE, Kavirayani A (2023). Intravenous anakinra for the treatment of haemophagocytic lymphohistiocytosis/macrophage activation syndrome: a systematic review. Eur J Haematol.

[7] Chen X, Fang J, Han P, Wu X, Han Y, Wang J, Li C (2026). Clinical characteristics and diagnostic indicators of childhood-onset systemic lupus erythematosus complicated with macrophage activation syndrome. Clin Rheumatol.

[8] Liu Y, Pan Y, Jin J, Wang P, Zhang T, Fan Z, Yu H (2024). Increased ferritin, serum lactate dehydrogenase, and aspartate aminotransferase levels predict macrophage activation syndrome complicating systemic lupus erythematosus: a retrospective study. Front Pediatr.

[9] Ravelli A, Minoia F, Davì S (2016). 2016 Classification Criteria for Macrophage Activation Syndrome Complicating Systemic Juvenile Idiopathic Arthritis: a European League Against Rheumatism/American College of Rheumatology/Paediatric Rheumatology International Trials Organisation Collaborative Initiative. Arthritis Rheumatol.

[10] Shakoory B, Geerlinks A, Wilejto M (2023). The 2022 EULAR/ACR points to consider at the early stages of diagnosis and management of suspected haemophagocytic lymphohistiocytosis/macrophage activation syndrome (HLH/MAS). Arthritis Rheumatol.

[11] Bilston L, Croden J, Taparia M, Karkhaneh M, Grossman J, Sun HL (2022). Validation of the HScore and the HLH-2004 diagnostic criteria for the diagnosis of hemophagocytic lymphohistiocytosis in a multicenter cohort. Eur J Haematol.

[12] George MR (2014). Hemophagocytic lymphohistiocytosis: review of etiologies and management. J Blood Med.

[13] Ho C, Yao X, Tian L, Li FY, Podoltsev N, Xu ML (2014). Marrow assessment for hemophagocytic lymphohistiocytosis demonstrates poor correlation with disease probability. Am J Clin Pathol.

[14] Baverez C, Grall M, Gerfaud-Valentin M (2022). Anakinra for the treatment of hemophagocytic lymphohistiocytosis: 21 cases. J Clin Med.

[15] Monteagudo LA, Boothby A, Gertner E (2020). Continuous intravenous anakinra infusion to calm the cytokine storm in macrophage activation syndrome. ACR Open Rheumatol.

[16] Fanouriakis A, Kostopoulou M, Andersen J (2024). EULAR recommendations for the management of systemic lupus erythematosus: 2023 update. Ann Rheum Dis.

